# Steroids from Marine-Derived Fungi: Evaluation of Antiproliferative and Antimicrobial Activities of Eburicol

**DOI:** 10.3390/md17060372

**Published:** 2019-06-21

**Authors:** Ana Camila Dos Santos Dias, Aurélie Couzinet-Mossion, Nicolas Ruiz, Fatima Lakhdar, Samira Etahiri, Samuel Bertrand, Lucie Ory, Christos Roussakis, Yves François Pouchus, El-Hassane Nazih, Gaetane Wielgosz-Collin

**Affiliations:** 1 Faculty of Pharmacy, University of Nantes, MMS-EA2160; 9, Rue Bias, 44000 Nantes, France; acamiladias@gmail.com (A.C.D.S.D.); aurelie.couzinet-mossion@univ-nantes.fr (A.C.-M.); nicolas.ruiz@univ-nantes.fr (N.R.); samuel.bertrand@univ-nantes.fr (S.B.); lucie.ory@etu.univ-nantes.fr (L.O.); yves-francois.pouchus@univ-nantes.fr (Y.F.P.); el-hassane.nazih@univ-nantes.fr (E.-H.N.); 2 Laboratory of Marine Biotechnology and Environment, Faculty of Science, University Chouaib Doukkali, BP 20 El Jadida, Morocco; fatima19862008@hotmail.fr (F.L.); setahiri@hotmail.com (S.E.); 3 Faculty of Pharmacy, University of Nantes, IICiMed, 9, Rue Bias, 44000 Nantes, France; christos.roussakis@univ-nantes.fr

**Keywords:** *Clonostachys rosea*, marine fungi, sterol, eburicol, cytotoxicity, antibacterial activity

## Abstract

The most common sterol in fungi is ergosterol, which has frequently been investigated in human pathogenic fungal strains. This sterol, and others isolated from fungal strains, has also demonstrated cytotoxicity against cancer cell lines and antimicrobial activities. Marine fungi can produce high amounts of bioactive compounds. So, a screening was performed to study sterol composition using GC/MS in 19 marine fungal strains and ergosterol was always the major one. One strain, *Clonostachys rosea* MMS1090, was selected due to its high amount of eburicol and a one strain many compounds approach was performed on seven culture media to optimize its production. After purification and structural identification by NMR, eburicol was assessed against four cancer cell lines, MCF-7, MDA-MB-231, NSCLC-N6-L16 and A549, and seven human pathogenic bacteria *Staphylococcus aureus*, *Bacillus* sp., *Bacillus cereus*, *Listeria ivanovii, Escherichia coli*, *Citrobacter freundii* and *Salmonella* spp. The most significant activity was cytotoxicity against MCF-7 cells (2 µM). This is the first report of such an accumulation of eburicol in the marine fungal strain *C. rosea* confirming its potential in the production of bioactive lipids.

## 1. Introduction

Lipids are actively involved in metabolic reactions like cell recognition, trans-membrane signaling, apoptosis, and growth or cell differentiation [1]. Among them, sterols are essential for the organization and functioning of eukaryotic organisms since they are components of cell membranes and they determine the permeability and fluidity of the membrane, therefore playing a crucial role in this dynamic [2,3,4]. The presence of sterols in every eukaryotic organism highlights the importance of these molecules in biological evolution [5,6]. Although sterols are present in all organisms, a great diversity can be observed depending on the kingdom studied. For example, within fungi, ergosterol is the main sterol which is present in the plasma membrane, unlike animals, in which it is cholesterol [7,8]. Biosynthetic pathways of those two sterols are distinct but have squalene as a common precursor. It is well known that sterols are studied for their importance for human nutrition [9]. Over the last few decades, numerous studies have described sterols, or more largely steroids, as bioactive compounds with antibacterial and anti-inflammatory activities or cytotoxicity against human cancer cells [10,11,12,13,14,15,16,17]. For example, a 24-branched Δ5 sterol isolated from a red seaweed was described as having antibacterial activity [18].

The exploration of microorganisms as sources of new bioactive compounds has a much more recent history in comparison to the use of plants or plant extracts in human medicine. However, the large number and variety of therapeutic agents isolated from microbial origins and used to treat human diseases have greatly contributed to the improvement of human health during the past century [19,20,21]. Among microorganisms, steroid compounds isolated from marine fungi have demonstrated bioactivities such as cytotoxicity against cancer cell lines [22,23]. For example, a new sterol, ergosta-8(14),22-diene-3,5,6,7-tetraol(3β, 5α, 6β, 7α, 22*E*), isolated from a marine *Penicillium* sp., exhibited cytotoxicity with IC_50_ values of 23 μM against human liver cancer cell (Hep G) [11].

The present investigation was undertaken to study the sterol composition of 19 marine fungal strains and then to assess their biological activities. Antimicrobial tests were performed against four Gram-positive bacteria, namely *Staphylococcus aureus*, *Bacillus* sp., *Bacillus cereus*, *Listeria ivanovii* and three Gram-negative ones, *Escherichia coli*, *Citrobacter freundii* and *Salmonella* spp. For the cytotoxic activity, two breast and two lung cancer cell lines were chosen, namely MCF-7, MDA-MB-231, NSCLC-N6-L16 and A549.

## 2. Results and Discussion

### 2.1. Marine Fungal Strains Screening

To explore sterol chemodiversity on marine strains, a screening was performed on Dextrose Casein Agar (DCA) culture media with 19 marine fungal strains. They were selected from the fungal collection of the laboratory either because they are representative of the major genera (*Trichoderma*, *Acremonium*, *Penicillium*) of the *Mer Molécules Santé* (MMS) marine fungal strain collection or because an unusual biological active conjugated fatty acid was previously isolated from the strain (*Clonostachys rosea* MM1090) [24].

The total lipid (TL) content of all studied strains are reported in Table 1.

In *Trichoderma* strains, the TL content varied from 2.2% (MMS1541) to 17.3% (MMS852). The same variability for TL content was observed in strains belonging to *Penicillium* genus with values ranging from 2.7% (MMS460) to 33.5% (MMS646). Higher TL contents were measured in the case of *Acremonium* species in comparison to *Trichoderma* and *Penicillium* strains; they varied from 7.3% (MMS540) to 50.7% (MMS889). Concerning *C. rosea* MMS1090, TL content was 21.4%. These values are in agreement with previous studies on lipid production, even though lipid content can be improved by the optimization of cultures conditions (composition, carbon source, pH and temperature) [25,26,27,28,29,30].

**Scheme 1:**
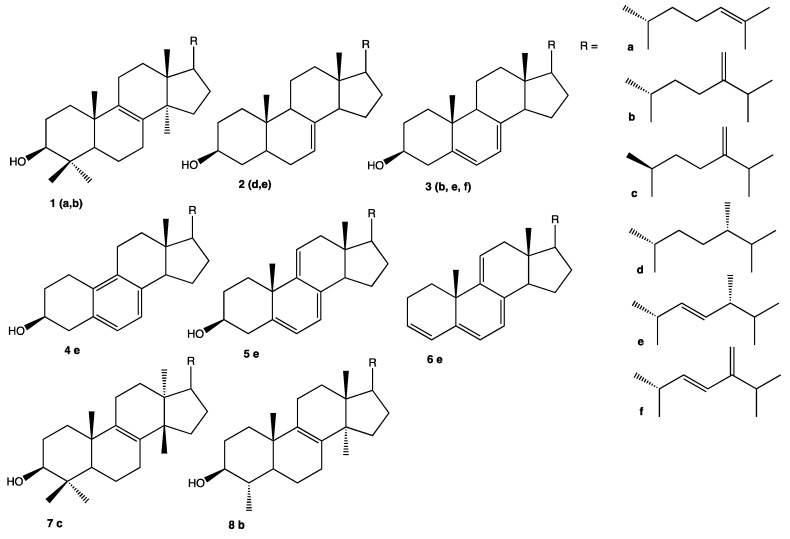
Structure of the steroid compounds.

After fungal biomass extraction, TL were saponified and sterols were identified by gaz chromatography coupled with mass spectrometry (GC-MS) based on a spectral data comparison with the literature (Table 1 and Scheme 1). The relative amounts were calculated using the percentage of peak area on chromatograms. Three major peaks were observed revealing a low complexity GC-MS profile of sterols produced by these strains. The main sterol observed in all unsaponifiable fractions was ergosterol (**3e**) (*m*/*z* 396 [M]^+●^) with contents ranging from 26.5% (MMS646) to 77.2% (MMS13) which represents a high variability but is in agreement with the literature [7]. In most strains (16/19), the second most abundant sterol was ergostatetraenol (**3f**) (ergosta-5,7,22,24(28)-tetraen-3β-ol, *m*/*z* 394 [M]^+●^) with contents ranging from 2.2% (*Acremonium* sp. MMS700) to 12.8% (*Trichoderma* sp. MMS1541). This compound is known to be a precursor of ergosterol [31]. The third most abundant compound was ergostapentaene (**6e**) (ergost-3,5,7,9(11),22-pentaene, *m*/*z* 376 [M]^+●^), with an amount ranging from 3.2% (*Acremonium* sp. MMS700) to 11.3% (*Acremonium* sp. MMS540). This compound was already observed in many studies of fungal sterols [17,32]. Neoergosterol (**4e**) (19-Norergosta-5,7,9,22-tetraen-3β-ol), *m*/*z* 380 [M]^+●^) was also observed in all fungal strains, except *C. rosea* MMS1090 where it was not detected. Its content ranged from 2.4% (*Trichoderma* sp. MMS13) to 4.9% (*Trichoderma* sp. MMS1541). Neoergosterol was reported to be a metabolite of ergosterol [33,34]. A fifth sterol was also observed in 13 out of the 19 strains studied corresponding to a C31:2 sterol with *m*/*z* 440 [M]^+●^, most probably eburicol (**1b**). It was a very minor compound (< 1%) for three *Penicillium* strains (MMS42, MMS330 and MMS460), two *Trichoderma* (MMS639 and MMS755) and two *Acremonium* ones (MMS700 and MMS713); and detected in trace amount (< 0.5%) in the case of *T. citrinoviride* MMS19. Its maximum content was observed in the cases of *C. rosea* MMS1090 (9.4%) where this content was unusual as it represented the second most abundant sterol of this strain. Eburicol content has been mostly studied in pathogenic strains when treated with antifungals [35]. To the best of our knowledge, only one study has investigated the cytotoxicity of eburicol against KB cells and multi-drug resistant strain KB-VIN cells, but no growth inhibition was observed at 20 µM [14]. *C. rosea* MMS1090 was chosen to purify and thus assess the biological potential effects of eburicol.

### 2.2. OSMAC Approach: Eburicol Production

To explore further eburicol production by *C. rosea* MMS1090 and optimize its culture conditions, a one strain many compounds (OSMAC) approach [36] was performed using seven culture media. The variation of the unsaponifiable content was assessed in the case of all culture media (Table 2). The results obtained indicated the ability of *C. rosea* MMS1090 to use the different available nutrients provided in the culture media leading to some differences in sterol production. A low variability of sterol content was observed (values from 7.5%—Kohlmeyer solid (KMS) and potato dextrose agar (PDA)—to 12.5%—yeast extract sucrose (YES)). Interestingly, as previously reported, the highest lipid production was obtained using the YES medium [24]. The high sterol content using YES medium could be explained by the high sucrose content of these particular culture medium (150 g/L), which will engage the lipid cells accumulation. In fact, under suitable culture conditions, some microorganisms may convert the carbon sources from several substrates into storage lipid, from which neutral lipids may represent about 90% of total cumulated lipids [29,37,38]; neutral lipids being mainly triglycerides, free fatty acids and sterols. Cultures conditions such as pH, temperature, carbon source and *C*/*N* ratio may change lipid production and composition [26,28,30,39,40]. These neutral lipids have a real importance in fungal growth and their content depends on the physiological state of the fungal culture.

Ergosterol remains the most abundant sterol produced by *C. rosea* MMS1090 whatever the culture conditions. The highest ergosterol contents were obtained on PDA and malt extract agar (MEA) media, 80.9% and 72.4%, respectively, and the lowest values were observed on KMS and YES culture media, 61.5% and 61.8%, respectively. The highest eburicol content was observed in extracts from *C. rosea* MMS1090 on YES culture medium, 11.7% of the total unsaponifiable. Lowest values were observed in extracts obtained from cultures on PDA and galactose casein agar (GCA) media, 2.3% and 2.7%, respectively.

**Figure 1:**
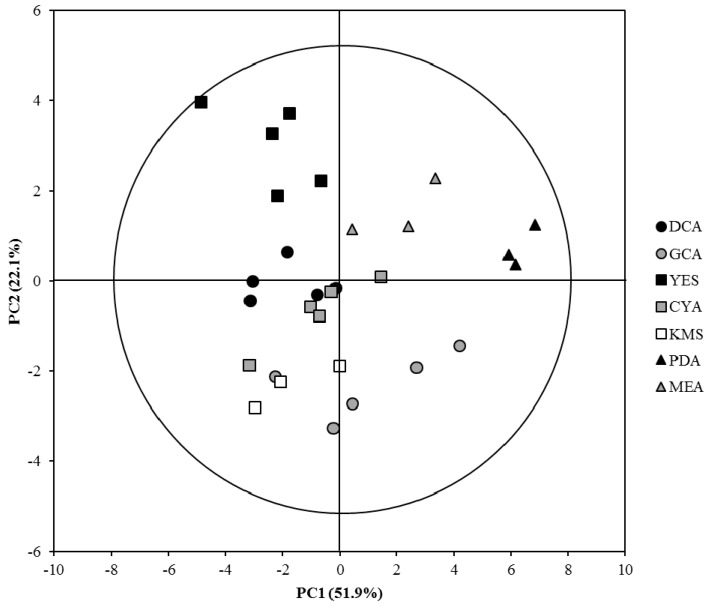
Principal component analysis (Pareto scaling) of unsaponifiable composition of *C. rosea* extracts according to culture medium.

To get a more comprehensive view of the data, unsaponifiable compositions were analyzed by principal component analysis (PCA) (Figure 1). The first two axes already represent accurately the data variability as they explain 74% of total variance. Interestingly, based on the unsaponifiable composition, all extracts cluster according to culture medium. This emphasizes the link between culture medium composition and active biosynthetic pathway in *C. rosea* [24,30]. The exploration of the loadings responsible for such clustering indicates specific unsaponifiable composition for some medium: (1) YES medium extract contains a higher amount of eburicol and lanosterol (**1a**); (2) PDA medium extract contains a higher amount of ergosterol; (3) KMS and GCA medium extracts contain a higher level of ergosta-5,7,24(28)-trienol (**3b**); and (4) KMS and CYA (Czapek concentrate Agar) medium extracts contain a higher level of 9-dehydroergosterol (**5e**) and ergostapentaene. What is noteworthy is that eburicol accumulation in fungi is principally reported in pathogenic fungi treated with azole compounds. Indeed, eburicol is the principal substrate to CYP51, an enzyme which promotes 14α-demethylation in sterols and which is inhibited by azole compounds. In this case, eburicol accumulation and ergosterol depletion therefore disrupt membrane organization and prevent further fungal growth [35,41,42,43].

### 2.3. Structural Identification of Eburicol

Two isomers are reported in the literature, eburicol and euphorbol (**7c**) and even by using GC-MS analyses, it is still not possible to identify which isomer is produced by *C. rosea* MMS1090. Therefore, a large-scale culture *C. rosea* MMS1090 was achieved using the YES culture medium to isolate and identify the observed isomer. After complete purification, the compound was observed as a white powder and the identification of the C31:2 sterol (*m*/*z* 440) was achieved by analysis of spectral data (GC-MS, HR-ESI-MS, and ^1^H and ^13^C NMR), melting point (mp ~155 °C) and a literature comparison.

**Figure 2:**
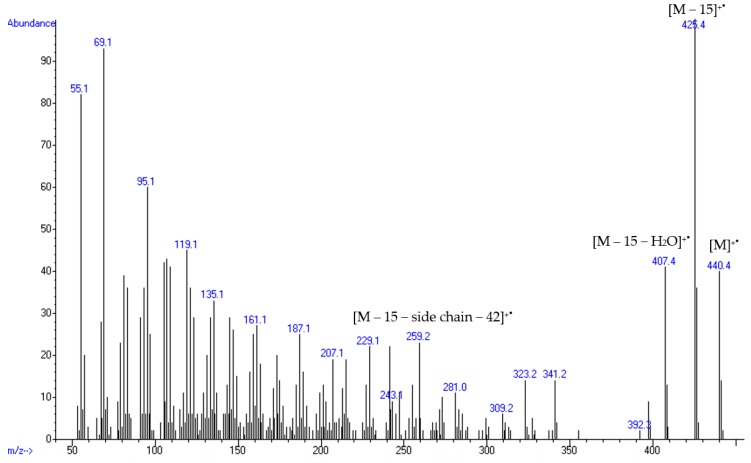
Gaz chromatography- mass spectrometry (GC-MS) spectrum of the C31:2 free sterol isolated from *C. rosea* MMS1090.

Initial identification was achieved using GC-MS spectrum (Figure 2). The ion at *m*/*z* 425 [M − 15]^+●^, is characteristic for the presence of a 14α-methyl group in a Δ8-sterol. The ion at *m*/*z* 259 [M − 15 − side chain − 42]^+●^ corresponded to a 24-methylene side chain and was in agreement with a C_9_H_17_ and a loss of the 14α-methyl group [44].

**Scheme 2:**
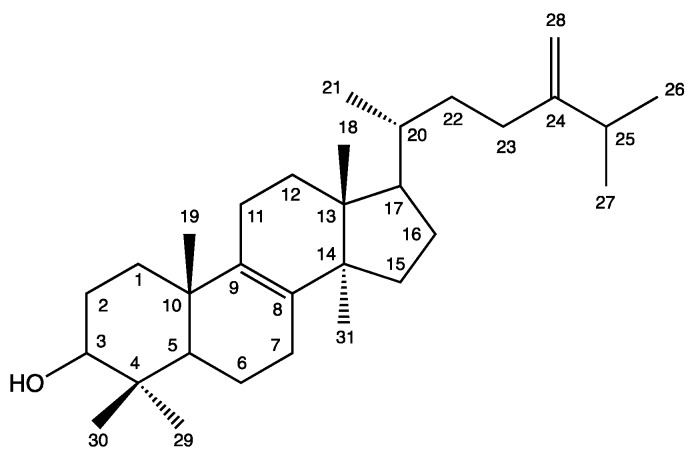
Eburicol structure.

Concerning the NMR analyses (Table 3, Figures S1–S6, Supplementary information), the singlet methyl signal at δ0.89 corresponded to methyl group (31-CH_3_) branched in C-14. A doublet signal at δ4.67 (*J* = 1,2 Hz) and the broad singlet at δ4.72 indicated the two C-28 protons, characteristic of a geminal coupling. Doublets were also observed at δ0.93 (*J* = 6.4 Hz), δ1.04 (*J* = 6.9 Hz) and δ1.03 (*J* = 6.9 Hz) for the C-21, C-26 and C-27 methyl groups. The ^1^H NMR signals were diagnostic for sterols with a 14α-methyl and 8(9)- and 24(28)-diene structure [45]. Analysis of the ^13^C NMR spectral data for the C31:2 isolated sterol showed signals in agreement with those for a 24-methylenelanost-8-en-3β-ol and were in agreement with shifts reported for eburicol in the literature (Scheme 2) [45,46,47].

The molecular formula of eburicol, C_31_H_53_O, was deduced by the ion detected at *m*/*z* 463.3908 [M + Na]^+^ in HR-ESI-MS (calculated for C_31_H_52_ONa [M + Na]^+^: 463.3910). The melting point (mp ~155 °C) was in accordance with those reported in the literature for eburicol [45]; with the euphorbol isomer having an mp at around 123–126 °C [48]. It is important to note that eburicol is the isomer generally found in fungi as it is a precursor of ergosterol [35] and euphorbol is mostly present in plants [49].

### 2.4. Biological Activities of Eburicol

#### 2.4.1. Antiproliferative Activity of Eburicol against Human Cancer Cells

Until now, several studies have been carried out on the cytotoxicity of sterols, especially ergosterol and its derivatives [17,22,50]. Therefore, eburicol was tested against breast cancer cell lines (MCF-7 and MDA) and lung cancer cell lines (NSCLC-N6-L16 and A549) to assess their antiproliferative activity using MTT assays (Table 4).

Eburicol exhibited cytotoxic activities on the four human cancer cell lines with IC_50_ lower than 40 µM. The most important activity was observed against the largely studied breast cancer cell line MCF-7 with IC_50_ at 2 µM which is the lowest for a sterol compound compared to literature. It should be noted that obtusifoliol (**8b**), although having a structure closely related to eburicol (a 4-methyl group instead of a 4,4-dimethyl group), exhibited a lower cytotoxic activity against MCF-7 and MDA-MB-231 cell lines [51].

On the other hand, among 10 ketone sterols isolated from a marine-derived fungi *Rhizopus* sp., the cytotoxic activity on another largely studied lung cancer cell line A549 was stronger (IC_50_ 4.9 µM) for 3β,15α-dihydroxyl-(22*E*,24*R*)-ergosta-5,8(14),22-trien-7-one [22]. This compound also demonstrated activity against two other cancer cell lines (P388: IC_50_ 1.4 µM and HL-60: IC_50_ 0.3 µM). More recently, new highly oxygenated sterols isolated from *Cladosporium* sp., a marine sponge-derived fungus, were inactive against the three selected tumor cell lines used in the study (MCF-7, K562, and SGC-7901) [55].

#### 2.4.2. Antimicrobial Activity of Eburicol against Human Pathogenic Bacteria

Bacterial infections remain a significant threat to human health. Due to the emergence of widespread antibiotic resistance, development of novel antibiotics is required in order to ensure that effective treatment remains available. Therefore, the antimicrobial activity of eburicol was also assessed against human pathogenic bacteria *B. cereus*, *Bacillus* sp., *L. ivanovii*, *S. aureus* (Gram-positive bacteria) and *C. freundii*, *E. coli*, *Salmonella* spp. (Gram-negative bacteria). A preliminary study was realized using the inhibition diameter (Table S1). Generally, a weak antimicrobial activity was observed against all bacteria with the highest inhibition zone (24 mm) for eburicol observed at 100 µg/mL on *S. aureus*. The minimum inhibitory concentration (MIC) was only determined against this strain and the value obtained was 62.5 µg/mL (142 µM).

To compare with other natural steroid compounds, ergosterol, the major sterol in lots of fungi, exhibited an MIC > 252 µM against a methicilin-resistant *S. aureus* (MRSA) [16]. Moreover, lanostane triterpenes isolated from *Fomitopsis rosea* demonstrated only weak antibacterial activity against a *S. aureus* strain, at 400 µg/mL with an inhibition zone from 14 to 25 mm [56]. Moreover, phytosterols, for example, sitosterol, campesterol and stigmasterol, with a Δ5 unsaturation, are known to present antibacterial activity against *S. aureus*, *S. albus*, *Streptococcus viridans*, *E. coli*, *Pseudomonas pyocyanea*, and *Klebsiella* [57]. Among Δ5 sterols, the most active one is the 24-propylidenecholest-5-en-3β-ol isolated from *Laurencia papillosa* red seaweed which shows an antibacterial activity against *S. aureus* ATCC25923 (MIC = 0.53 µg/mL) [18]. Only two publications reported antibacterial activity of Δ7 steroids against *S. aureus* ATCC 6538 with ergosta-7,22-dien-3-one and ergosta-7,22-dien-3β-ol, which did not demonstrate any activity [58] while 24-methyl-5α-cholesta-7,22-dien-3β-ol and 24-methyl-5α-cholest-7-en-3β-ol exhibited a light one, with a MIC of 2 mg/mL on *S. aureus* ATCC 25923 strain sensitive to penicillin and tetracycline [59]. In the best of our knowledge, any activity has been reported before for a 4,4-dimethyl sterol or a Δ8 sterol.

## 3. Materials and Methods

### 3.1. Marine Fungal Strains

Fungal strains were isolated from shellfish farming areas of the French Atlantic west coast. Fungal identifications were performed on the basis of their morphological and microscopic features and were completed for 11 strains by sequencing the internal transcribed spacers ITS1 and ITS2 and the 5.8S regions and deposited in GenBank. The strains were conserved in the fungal collection of the MMS laboratory of the University of Nantes under the reference MMS + number. Nineteen strains from four genera were selected: *Trichoderma harzanium* MMS13 (access number: JQ653065); *T. citrinoviride* MMS19 (JQ608473); *T. reesei* MMS510 (GU947793); *T. atroviride* MMS639 (JQ653056); *T. capilare* MMS755 (JQ653068); *T. capilare* MMS852 (JQ653070); *T. pleuroticola* MMS1541 (JQ653064); *Acremonium* sp. MMS540; *Acremonium* sp. MMS594; *Acremonium* sp. MMS700; *Acremonium* sp. MMS713; *Acremonium* sp. MMS862; *Acremonium* sp. MMS887; *Acremonium* sp. MMS889; *Penicillium expansum* MMS42 (JN794527); *P. ubiquetum* MMS330 (MH172366); *P. canescens* MMS460 (KU720405); *Penicillium* sp. MMS646; *Clonostachys rosea* MMS1090 (KR011118).

### 3.2. Culture Media Conditions

Petri dishes (10 cm diameter) were inoculated with conidia suspension (1 × 10^6^ cells/mL) on seven marine-like solid media prepared with artificial seawater (Reef Crystals 36 g/L): **DCA** (Dextrose 40 g/L, enzymatic digest of casein 10 g/L, agar 15 g/L, Difco); **CYA** (Czapek concentrate 10 mL, yeast extract 5 g/L, K_2_HPO_4_ 1 g/L, trace metal solution 1 mL, sucrose 30 g/L, Agar 15 g/L); **YES** (yeast extract 20 g/L, sucrose 150 g/L, agar 20 g/L plus salts); **GCA** (galactose 40g/L, enzymatic digest of casein 10 g/L, agar 15 g/L); **KMS** (Kohlmeyer solid, dextrose 5 g/L, MgSO4, 7H_2_O 2.4 g/L, NH_4_NO_3_ 2.4 g/L Tris buffer 1.21 g/L, agar 15 d/L); **PDA** (potato extract 4 g/L, dextrose 20 g/L, ZnSO_4_, 7 H_2_O 0.01 g/L, CuSO_4_, 5 H_2_O 0.005 g/L, Agar 15 g/L); **MEA** (malt extract 20 g/L, peptone 1g/L, dextrose 20g/L, Agar 20 g/L). Cultures were incubated in natural light for 10 days at 27 °C.

### 3.3. Lipid Extraction and Saponification

For each culture media, the biomass on Petri dishes was scrapped off and removed using a scalpel and allowed to dry in an oven at 65 °C and weighed. Lipids were extracted from the dried biomass by two-step CH_2_Cl_2_/MeOH extraction 2:1 then 1:2 (v/v) during 2 h at room temperature. The resulting extracts were filtered, pooled and washed with water (0.08% KCl, 40% of total volume). Then the organic phase was evaporated to dryness under vacuum providing total lipid crude extracts (TL) which was expressed as a percentage of the total extracted dry biomass. Total lipids were then saponified with ethanolic KOH (2 M) at 80 °C under reflux for 1.5 h. After cooling, water and hexane were added, 1:2 (v/v), and the organic phase, containing sterols (unsaponifiable), was dried with anhydrous Na_2_SO_4_, filtered and the solvent was evaporated. The unsaponifiable fraction was analyzed by gas chromatography coupled with mass spectrometry (GC-MS, Hewlett Packard, Wilmington, DE, USA). A part of the unsaponifiable fraction was acetylated using acetic anhydride and pyridine and heated 1 h at 80 °C in order to obtain sterol acetates.

### 3.4. General Spectrometry Analysis

Free and acetylated sterols were analyzed using a GC-MS instrument (Hewlett Packard HP 6890—GC System, Wilmington, DE, USA) linked to a mass detector (HP 6890—E.I. 70 eV) equipped with a SLB-5^TM^ column (60 m × 0.25 mm × 0.25 µm). The carrier gas was helium at a flow rate of 1 mL/min. The temperatures of the injector and detector were respectively set at 250 °C and 280 °C. One microliter was injected in splitless mode. The initial temperature of the GC-oven was 200 °C with a subsequent increase (3 °C/min) up to 310 °C. The solvent delay was 9 min. The relative amount of each component was determined as the percent of the total ion current (TIC) as free compounds. Acetylated sterols were only used to identify the different molecules.

High resolution mass spectra were recorded using a Micromass Zab Spec Tof spectrometer (Waters Corporation, Milford, MA, USA) (positive mode, ion-source acceleration 4.5 kV, ion-source temperature 200 °C, methanol as solvent). ^1^H- and ^13^C-NMR spectra were obtained on a NMR Bruker Avance III 400 spectrometer (Ettlingen, Germany) with triple Probe TBI multinuclear in CDCl_3_ with tetramethylsilane as reference to an internal standard. Chemical shifts and coupling constants were expressed in δ (ppm) and Hz, respectively.

### 3.5. Production, Purification and Structural Elucidation of Sterol from C. rosea MMS 1090

For production of sterols, cultures of *C. rosea* MMS 1090 were performed on YES culture medium. Petri dishes (20 cm diameter, 125 mL) were incubated at 27 °C in natural light for 10 days. After biomass extraction, TL were separated as described previously [24]. Briefly, TL were separated in lipid classes on an open silica gel column (Kieselgel—60 Å—0.063–0.2 mm—ASTM). The crude extract from MMS1090 was separated in 20 fractions according to the eluents: D1 to D8 for dichloromethane fractions; A1 to A5 for acetone fractions; M1 to M7 for methanol fractions.

Lipid fractions composition was controlled by thin layer chromatography (TLC) performed on precoated silica gel F_254_ plates. After development, the dried plates were sprayed with 50% H_2_SO_4_-vanillin and orcinol reagents. Sterols were eluted from D3 to D8. The D3 fraction enriched in sterols was then purified by preparative TLC (hexane/diethyl ether/acetic acid: 50/50/0.75: v/v/v) in silica gel F_254_ plates. White amorphous powder (melting point ~155 °C) was obtained. Fraction purity was monitored by GC-MS analysis.

### 3.6. Cell Viability Assay

Human breast cancer MCF-7 and MDA-MB-231 cells were purchased from the European Collection of Animal Cell Cultures (ECACC, Salisbury, UK). Human lung cancer NSCLC-N6-L16 and A549 (reference CCL-185) cell lines were obtained from IICiMED laboratory (Nantes, France) and the American Type Culture Collection (ATCC). Cells were cultured at 37 °C in a humidified incubator with 5% CO_2_. MCF-7 cells were cultured in DMEM medium supplemented with 10% fetal bovine serum (FBS), 1% glutamine and 1% penicillin–streptomycin. NSCLC-N6-L16 and A549 cells were cultured in RPMI 1640 medium with 5% fetal calf serum, 100 IU penicillin/mL, 100 µg streptomycin/mL and 2 mM glutamine.

The four cancer cell lines were treated with different concentrations of the purified sterol for 72 h and cell growth was estimated by a colorimetric assay based on the reduction of tetrazolium dye (MTT, Sigma Aldrich, Saint Quentin Fallavier, France) to a blue formazan product by live mitochondria. Optical density corresponding to solubilized formazan was read at 570 nm. The relative cell viability was expressed as a percentage of the control that was not treated with sterol. Docetaxel was used as positive control for MCF-7 (IC_50_ = 2.7 nM) and for MDA-MB-231 (IC_50_ = 100 nM) and vinorelbine for A549 (IC_50_ = 0.13 µM) and NSCLC-N6-L16 (IC_50_ = 2.7 µM).

### 3.7. Antibacterial Activity against Human Pathogens

The strains used to evaluate the antimicrobial activity were obtained from the collection of the Pasteur Institut (CIP) and the ATCC. Gram-positive bacteria were *Staphylococcus aureus* (ATCC 25923), *Bacillus* sp. (CIP 104717), *Bacillus cereus* (ATCC 33019), and *Listeria ivanovii* (ATCC 19119). Gram-negative bacteria were *Escherichia coli* (ATCC 10536), *Citrobacter freundii* (ATCC 8090) and *Salmonella* spp. Antibacterial activity of purified sterol was assessed in triplicates using agar diffusion with cellulose disks of 6 mm in diameter, dissolved in a minimum volume of solvent (dichloromethane) and cellulose disks were imbibed with different concentrations. After solvent evaporation, the disks were placed on the surface of a Petri dish previously seeded with 5 mL of a bacteria suspension (0.2 × 10^4^ bacteria/mL) and incubated at 37 °C for 24 h. Two concentrations were tested (50 and 100 μg/mL). Disks with the streptomycin standard at the same concentrations were used as control. The results were obtained by measuring the diameter of inhibition zone for each disk and expressed in millimeter. The determination of the minimum inhibitory concentration (MIC) was carried out according to the microtitre technique described by Sarker et al. [60] with some modifications. Briefly, 100 μL of the eburicol were added and serial dilutions were performed to achieve final concentrations ranged from 0.24 µg/mL to 500 μg/mL. Then 100 μL of Mueller Hinton broth (BMH) inoculated with 100 μL of a *S. aureus* suspension are added to reach a concentration of 10^6^ CFU/mL. The antibacterial activity was examined after incubation at 37 °C for 18–24 h using resazurin (7-hydroxy-3H-phenoxazin-3-one) as colored indicator. BMH without bacterial suspension was used as negative control and streptomycin as positive one (MIC = 3.4 µM). All experiments were performed in triplicate.

### 3.8. Statistical analyses

Statistical analyses (principal component analyses) were performed using SIMCA-P 13^®^ (Umetrics, Umeå, Sweden).

## 4. Conclusions

This present study compared sterol production between 19 marine-sourced fugal strains. For 17 strains, the major steroid compounds were by order of abundance ergosterol, ergostatetraenol, ergostapentaene, neoergosterol and eburicol. However, an unusual sterol production profile was observed in the case of *C. rosea* MMS1090 where the second most abundant sterol was eburicol, after ergosterol. To optimize culture conditions and produce high amounts of eburicol an OSMAC approach was performed. The highest lipid and eburicol production was obtained on YES culture conditions. After its isolation and structural identification, eburicol biological activities were evaluated. The present results showed that eburicol had a weak antimicrobial activity, against *S. aureus* (but proved a significant cytotoxic effect against two breast human cancer cell lines, in particular against MCF-7 at 2 µM (IC_50_). To conclude, this study of fungal sterols confirmed the unusual profile of the marine-sourced *Clonostachys rosea* MMS1090 strains and confirms its potential in the production of bioactive lipids.

## Figures and Tables

**Table 1:** Total lipid (TL) content and relative unsaponifiable composition of the different strains screened (*n* = 1).

Genus	Strain	TL Content (% w/DW)	Relative Unsaponifiable Composition (% of Total Unsaponifiable Fraction) as Free Compounds				
			Ergostapentaene (6e) *m*/*z* 376 [M]^+●^	Ergostatetraenol (3f) *m*/*z* 394 [M]^+●^	Ergosterol (3e) *m*/*z* 396 [M]^+●^	Neoergosterol (4e) *m*/*z* 380 [M]^+●^	Eburicol (1b) *m*/*z* 440 [M]^+●^
*Trichoderma*	MMS13	13	4.8	4.5	**77.2**	2.4	ND
	MMS19	9	7.9	10.2	43.5	2.5	Tr
	MMS510	5.8	4.3	4.8	66.8	3.1	ND
	MMS639	12.9	5.1	5.5	63.8	3.5	0.7
	MMS755	8.8	5.1	10.7	50.8	3.9	0.8
	MMS852	17.3	6.5	3.1	79.2	4.3	ND
	MMS1541	2.2	6.0	**12.8**	22.8	**4.9**	1.5
*Mean*		*10 ± 5*	*6 ± 1*	*7 ± 4*	*54 ± 20*	*3.5 ± 0.9*	*0.5 ± 0.4*
*Acremonium*	MMS540	7.3	**11.3**	11.5	30.8	3.9	ND
	MMS594	18.1	5.4	9.3	44.7	3.5	2.1
	MMS700	11.4	3.2	2.2	65.5	3.8	0.6
	MMS713	9.2	3.3	6.5	66.3	3.5	0.5
	MMS862	8	5.1	8.8	59.9	4.1	2.4
	MMS887	20.1	7.4	8.9	56.1	3.2	5.4
	MMS889	**50.7**	5.0	7.3	73.3	4.1	ND
*Mean*		*18 ± 15*	*6 ± 3*	*8 ± 3*	*57 ± 15*	*3.7 ± 0.3*	*2 ± 2*
*Penicillium*	MMS42	5.7	4.0	5.5	60.3	3.1	0.6
	MMS330	2.9	6.1	7.6	49.3	3.4	0.7
	MMS460	2.7	5.8	7.0	48.1	2.8	0.8
	MMS646	33.5	5.1	8.3	26.5	2.8	ND
*Mean*		*11 ± 15*	*5.3 ± 0.9*	*7 ± 1*	*46 ± 14*	*3 ± 0.3*	*0.5 ± 0.1*
*Clonostachys*	MMS1090	21.4	6.6	5.5	66.2	ND	**9.4**

DW: dry weight; Tr: traces < 0.5%; N.D.: Not Detected.

**Table 2:** Unsaponifiable content of total lipids (% *w*/*w* of TL) and relative composition of the unsaponifiable fraction (% of unsaponifiable) from MMS 1090 based on the seven culture media (*n* = 3) as free compounds.

Culture Media	Unsaponifiable Content (% *w*/*w* of TL)	Relative Unsaponifiable Composition of MMS1090 Lipid Extract (% of Unsaponifiable Fraction) as Free Compounds									
		Ergostapentaene (6e)	9-dehydroergosterol (5e)	Ergostatrienol (3b)	Ergosterol (3e)	Ergostadienol (2e)	Neoergosterol (4e)	Fungisterol (2d)	Lanosterol (1a)	Eburicol (1b)	Others
DCA	8.8 ± 1.4	5.9 ± 0.6	5.0 ± 0.5	1.9 ± 0.2	63.2 ± 3.3	4.7 ± 0.6	2.2 ± 0.2	0.8 ± 0.2	Tr ^(1)^	**8.0 ± 1.3**	8.2 ± 1.1
GCA	9.5 ± 1.1	4.7 ± 0.9	5.3 ± 1.1	3.1 ± 0.4	68.2 ± 6.3	2.8 ± 0.3	2.5 ± 0.4	Tr ^(1)^	0.3 ± 0.2	**2.7 ± 0.8**	13.7 ± 2.0
YES	12.5 ± 2.1	3.8 ± 0.5	3.1 ± 0.7	1.1 ± 0.1	61.8 ± 3.4	4.2 ± 0.5	2.1 ± 0.2	0.8 ± 0.3	0.8 ± 0.3	**11.7 ± 2.4**	10.6 ± 1.2
CYA	10.6 ± 2.4	5.1 ± 1.2	5.5 ± 0.8	1.4 ± 0.5	65.1 ± 3.8	8.9 ± 1.3	1.8 ± 0.5	1.7 ± 0.1	ND ^(1)^	**5.6 ± 0.5**	4.9 ± 1.2
KMS	7.5 ± 3.5	4.7 ± 0.9	5.9 ± 0.7	2.8 ± 0.1	61.5 ± 3.8	3.6 ± 0.8	2.6 ± 0.7	1.3 ± 1.1	Tr ^(1)^	**3.81 ± 0.06**	12.7 ± 1.2
PDA	7.5 ± 1.8	3.3 ± 0.5	1.7 ± 0.3	0.9 ± 0.1	80.9 ± 1.3	5.4 ± 0.5	1.97 ± 0.02	0.6 ± 0.3	ND ^(1)^	**2.3 ± 0.6**	2.9 ± 0.4
MEA	10.0 ± 2.5	3.4 ± 0.3	3.6 ± 0.4	1.3 ± 0.2	72.4 ± 4.1	2.9 ± 0.4	2.0 ± 0.5	0.8 ± 0	Tr ^(1)^	**7.5 ± 0.8**	6.1 ± 1.3

(1) Tr: traces < 0.5%; ND: Not Detected.

**Table 3:** ^1^H NMR (400.13 MHz) and ^13^C NMR (100.61 MHz) in CDCl_3_ of eburicol.

Position	^1^H NMR δ (ppm)	m, *J* (Hz)	^13^C NMR δ (ppm)
1	1.35–2.34	*M*	35.6
2	1.35–2.34	*M*	27.8
3	3.24	*dd*, 4.7 and 11.5	79.0
4	-	-	38.6
5	1.35–2.34	*M*	50.4
6	1.35–2.34	*M*	18.2
7	1.35–2.34	*M*	28.2
8 and 9	-	-	134.4
10	-	-	37.0
11	1.35–2.34	*M*	21.0
12	1.35–2.34	*M*	26.5
13	-	-	44.5
14	-	-	49.8
15	1.35–2.34	*M*	31.3
16	1.35–2.34	*M*	31.0
17	1.35–2.34	*M*	50.4
18	0.70	*S*	15.7
19	0.99	*S*	18.7
20	1.35–2.34	*M*	36.5
21	0.93	*d,* 6,4	19.1
22	1.35–2.34	*M*	35.0
23	1.35–2.34	*M*	30.8
24	-	*-*	156.9
25	2.24	*M*	22.0
26	1.04	*d,* 6.9	33.8
27	1.03	*d,* 6.9	21.9
28	*4.67* and *4.72*	*d* and *s, 1.2*	105.9
29	0.82	*S*	15.4
30	1.01	*S*	27.9
31	0.89	*S*	24.3
OH	5.35	*m*	

**Table 4:** Cytotoxic activities of eburicol compared to literature.

Sterol Compounds	IC_50_ (µM)				Reference
	MCF-7	MDA-MB-231	NSCLC-N6-L16	A549	
Eburicol	**2.0** ± 0.7	15.7 ± 0.9	24.5 ± 0.5	38 ± 3	Present study
Obtusifoliol	29.3	41.8	-	-	[51]
Gorgosterol	71.2	-	-	-	[52]
4-methyl sterol	-	-	-	20.4	[53]
Ketone sterols	-	-	-	**4.9** to >100	[22]
Hydroperoxysterol	20.5	-	-	40.4	[54]
Polyhydroxysterols	10.6–71.9	-	-	-	[52]

## Supplementary Materials

The following are available online at https://www.mdpi.com/1660-3397/17/6/372/s1, Figure S1: ^1^H NMR spectrum of Eburicol in CDCl_3_ (400 MHz, 25 °C); Figure S2. COSY spectrum of Eburicol in CDCl_3_ (400 MHz, 25 °C); Figure S3. Expanded HSQC spectrum of eburicol in CDCl_3_ (400 MHz for ^1^H, 25 °C); Figure S4. Expanded HSQC spectrum of eburicol in CDCl_3_ (400 MHz for ^1^H, 25 °C); Figure S5. Expanded HMBC spectrum of eburicol in CDCl_3_ (400 MHz for ^1^H, 25 °C); Figure S6. Expanded HMBC spectrum of eburicol in CDCl_3_ (400 MHz for ^1^H, 25 °C); Table S1. Evaluation of antimicrobial activity of eburicol against human pathogenic bacteria (*n* = 3). Values should be compared to the standard Streptomycin results (12 ± 2 at 50 µg/mL and 15 ± 2 at 100 µg/mL for all strains).
