# Supplementary material for: Steroids from Marine-Derived Fungi: Evaluation of Antiproliferative and Antimicrobial Activities of Eburicol
Source: Mar Drugs. 2019 Jun 21;17(6):372. doi: 10.3390/md17060372 (PMC6628047; doi:10.3390/md17060372)
Supplement: Supplementary file 1 [file marinedrugs-17-00372-s001.pdf]

Supplementary Materials

Steroids from Marine-Derived Fungi: Evaluation of Antiproliferative and Antimicrobial Activities of Eburicol

Ana Camila dos Santos Dias, Aurélie Couzinet-Mossion, Nicolas Ruiz, Fatima Lakhdar, Samira Etahiri, Samuel Bertrand, Lucie Ory, Christos Roussakis, Yves-François Pouchus, Hassan Nazih, Gaetane Wielgosz-Collin

Table of Contents

Figure S1. ^{1}H NMR spectrum of Eburicol in $CDCl_3$ (400 MHz, 25°C)

Figure S2. COSY spectrum of Eburicol in $CDCl_3$ (400 MHz, 25°C)

Figure S3. Expanded HSQC spectrum of eburicol in $CDCl_3$ (400 MHz for ^{1}H, 25°C)

Figure S4. Expanded HSQC spectrum of eburicol in $CDCl_3$ (400 MHz for ^{1}H, 25°C)

Figure S5. Expanded HMBC spectrum of eburicol in $CDCl_3$ (400 MHz for ^{1}H, 25°C)

Figure S6. Expanded HMBC spectrum of eburicol in $CDCl_3$ (400 MHz for ^{1}H, 25°C)

Table S1. Evaluation of antimicrobial activity of eburicol against human pathogenic bacteria (n = 3). Values should be compared to the standard **Streptomycin** results (**12 ± 2 at 50 µg/mL** and **15 ± 2 at 100 µg/mL** for all strains).

Figure S1. ^{1}H NMR spectrum of Eburicol in $CDCl_3$ (400 MHz, 25°C)

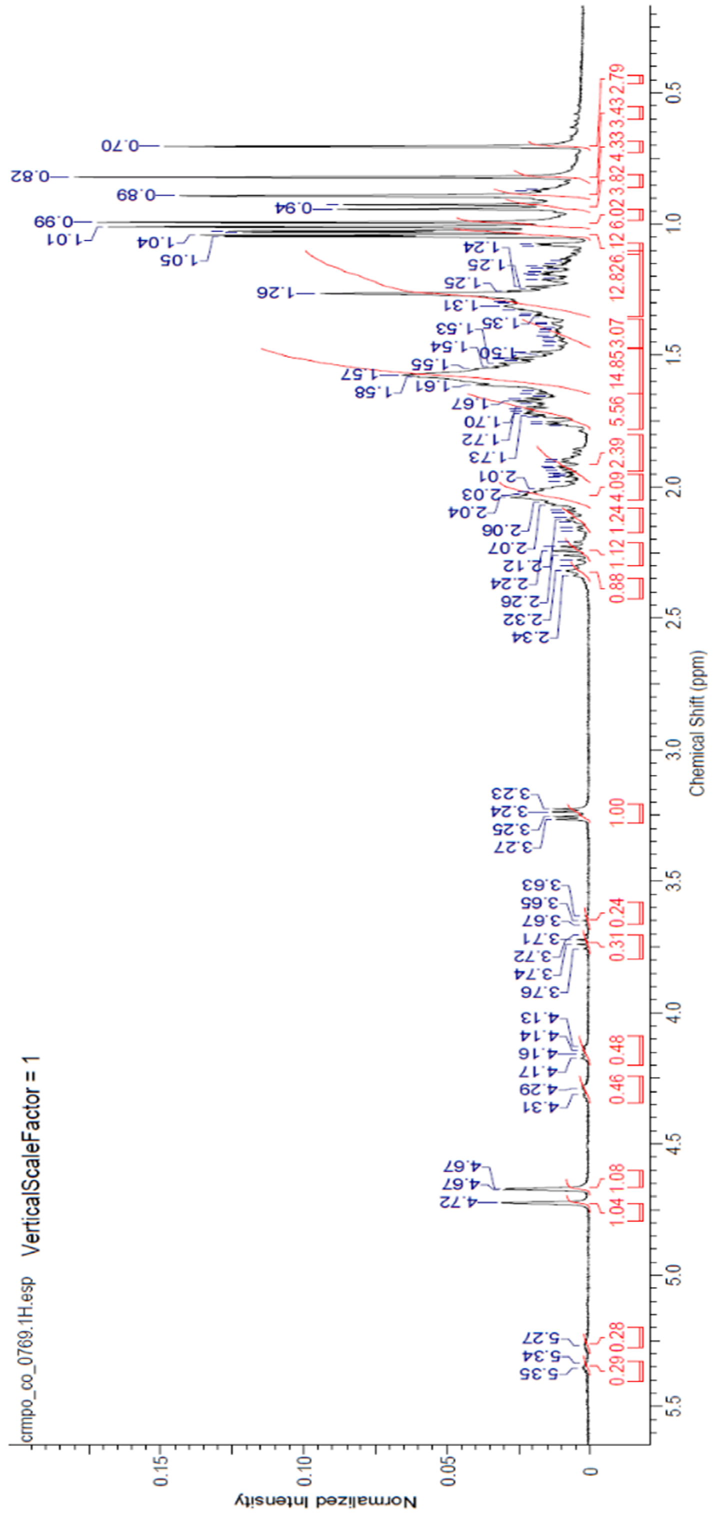

Figure S2. COSY spectrum of Eburicol in $CDCl_3$ (400 MHz, 25°C)

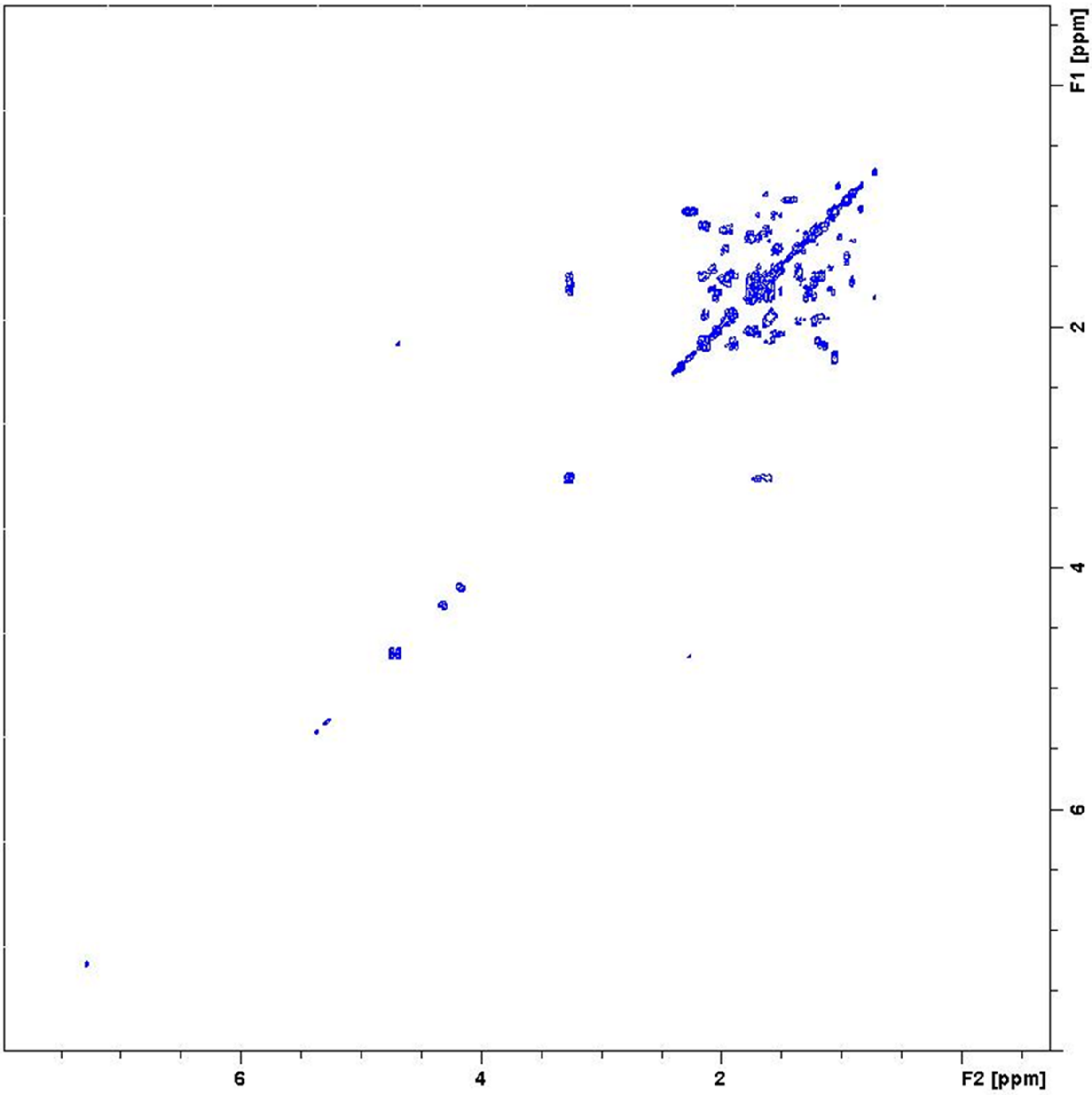

Figure S3. Expanded HSQC spectrum of eburicol in $CDCl_3$ (400 MHz for ^1H, 25°C)

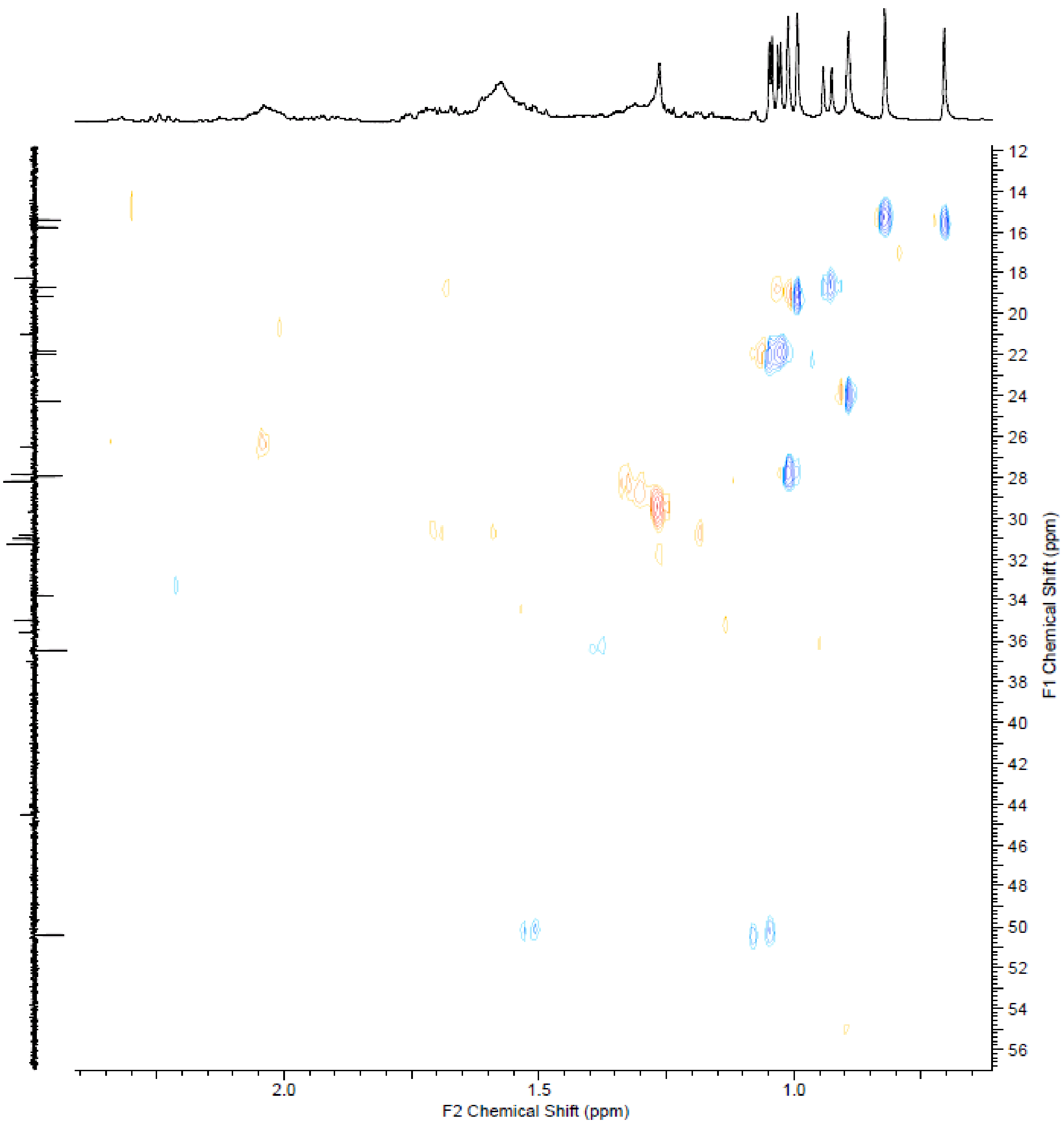

Figure S4. Expanded HSQC spectrum of eburicol in $CDCl_3$ (400 MHz for ^1H, 25°C)

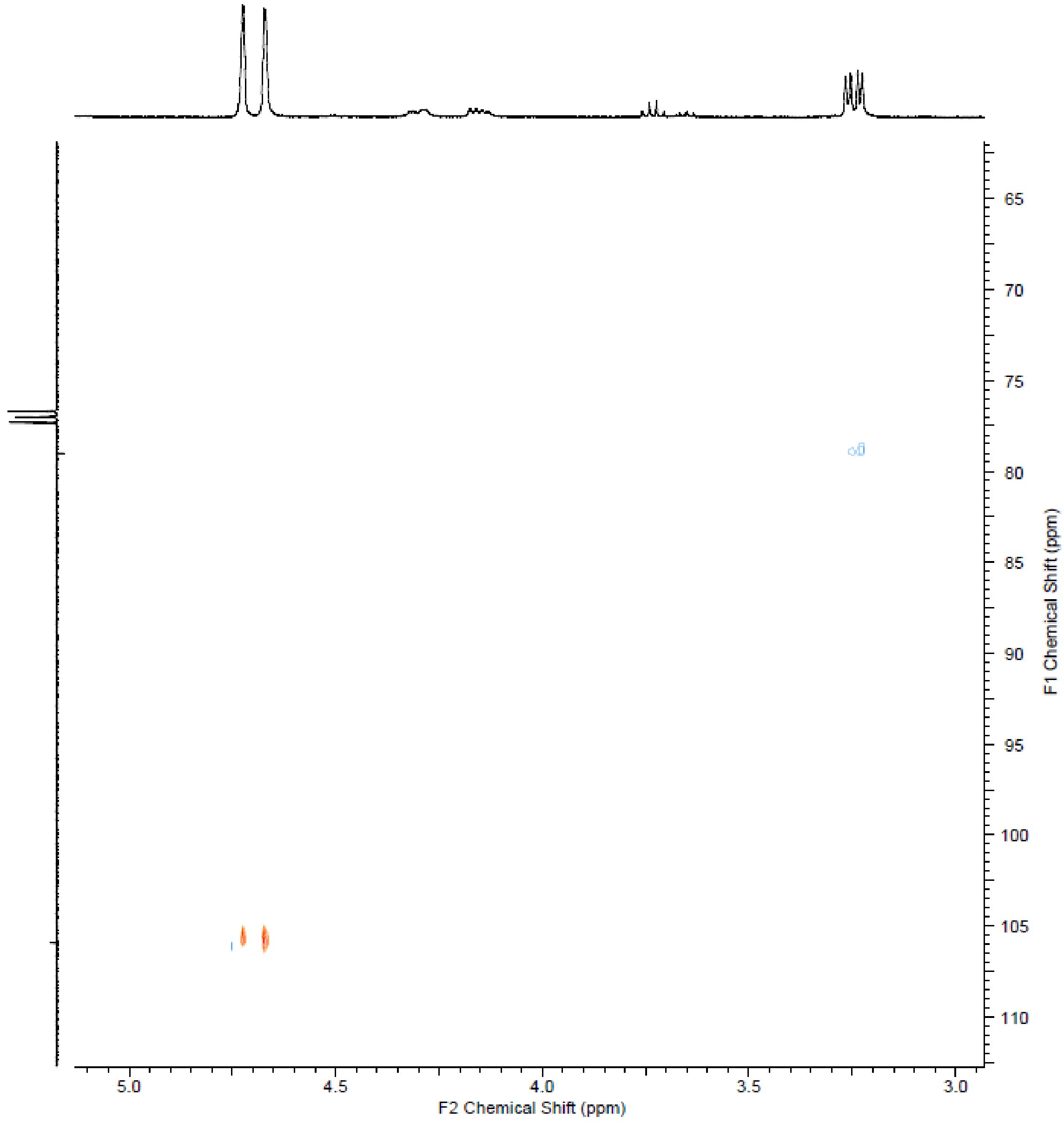

Figure S5. Expanded HMBC spectrum of eburicol in $CDCl_3$ (400 MHz for ^1H, 25°C)

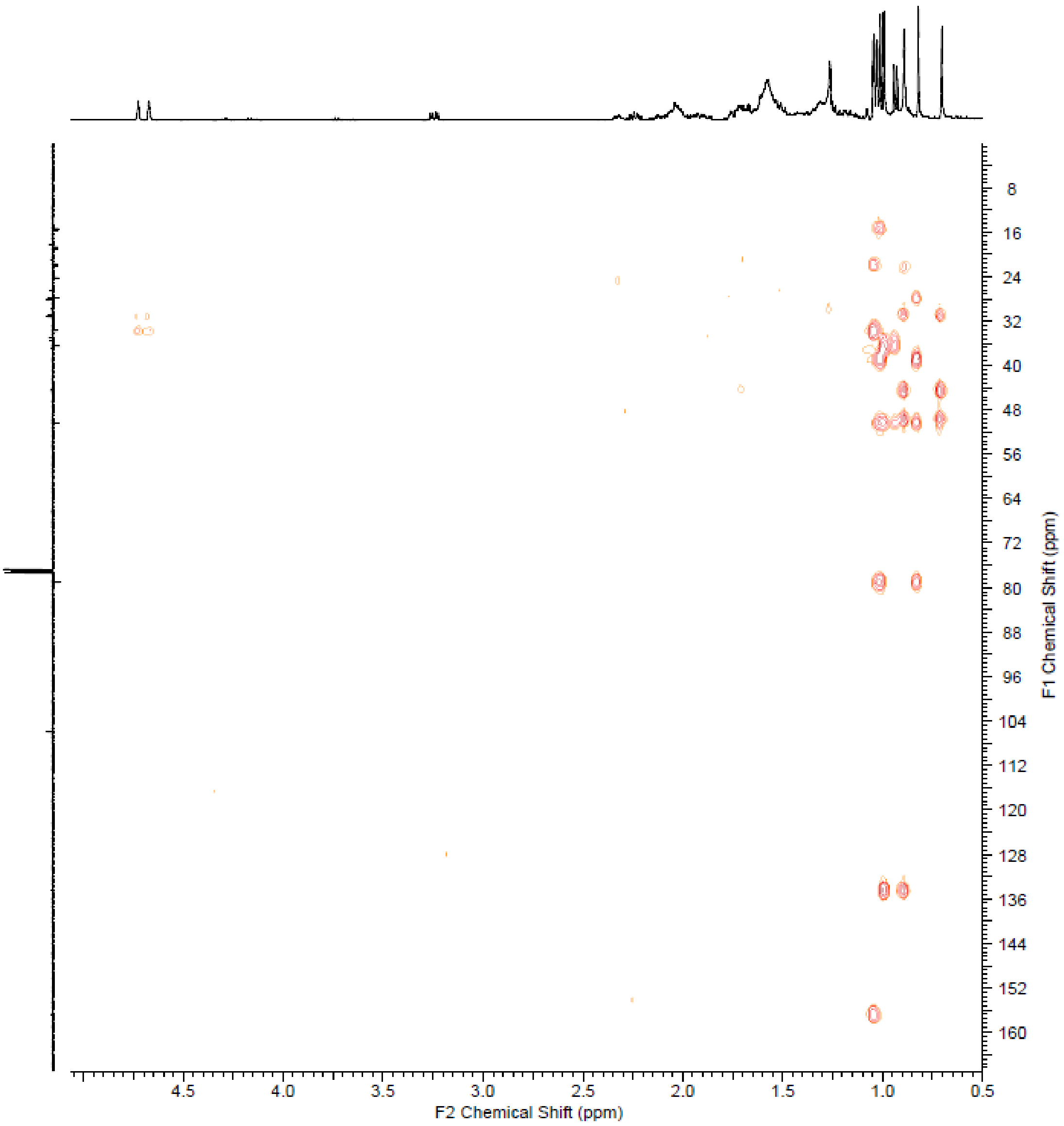

Figure S6. Expanded HMBC spectrum of eburicol in $CDCl_3$ (400 MHz for ^1H, 25°C)

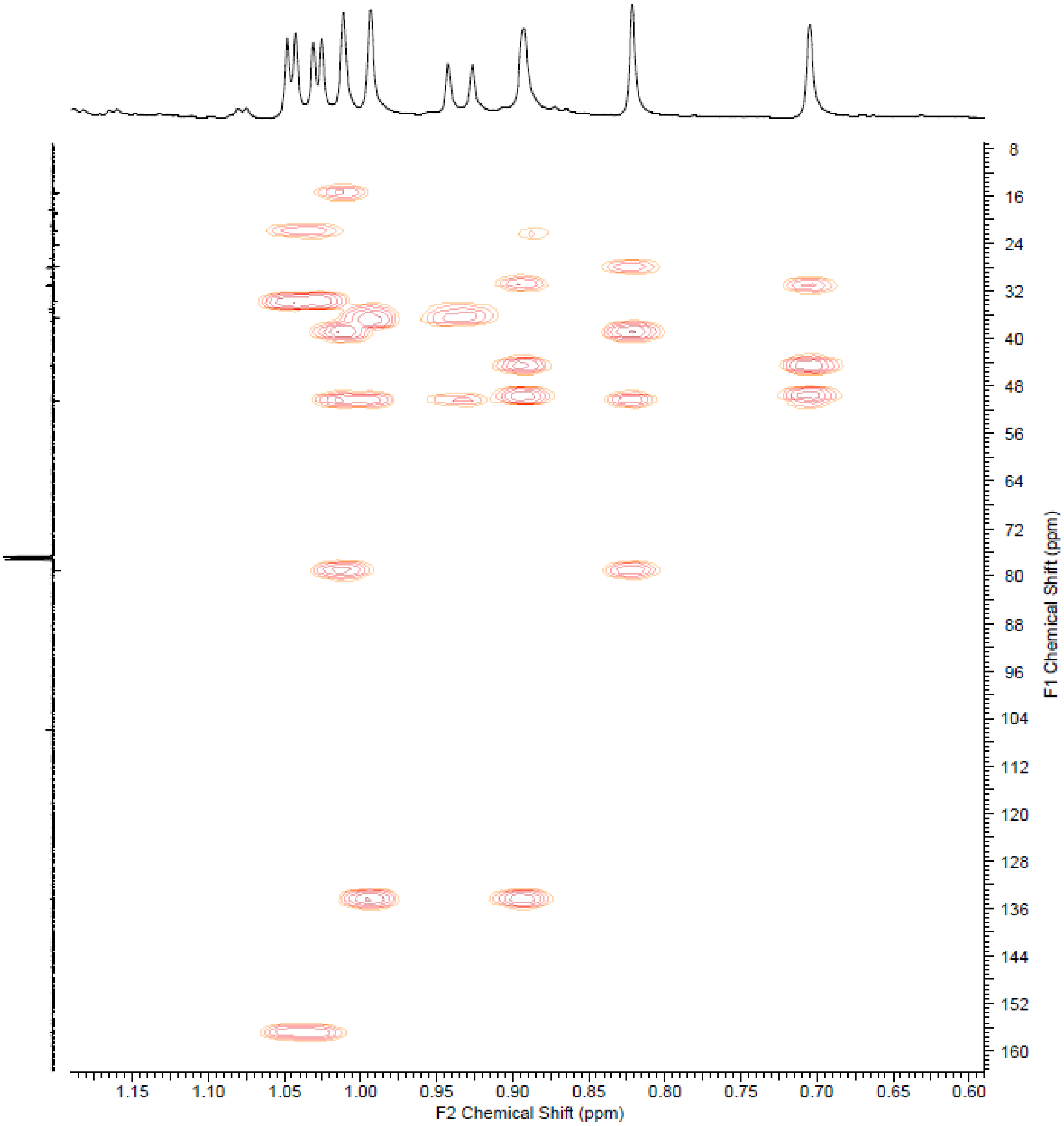

Table S1. Evaluation of antimicrobial activity of eburicol against human pathogenic bacteria (n = 3). Values should be compared to the standard **Streptomycin** results (**12 ± 2 at 50 μg/mL** and **15 ± 2 at 100 μg/mL** for all strains).

**Strains**	**Inhibition zone (mm)[(1)] after treatment with eburicol**	
	**50 μg/mL**	**100 μg/mL**
B. cereus	10 ± 1	11 ± 1
Bacillus sp.	8 ± 0	12 ± 1
L. ivanovii	10 ± 1	9 ± 1
S. aureus	7 ± 0	**24 ± 4**
C. freundii	8 ± 1	11 ± 2
E. coli	10 ± 1	ND
Salmonella spp.	9 ± 1	8 ± 2

(1): Ø ≤ 8mm: no significant antimicrobial activity; 8 < Ø ≤ 12 mm: weak antimicrobial activity; 12 < Ø ≤ 14 mm: significant antimicrobial activity; Ø > 14 mm: high antimicrobial activity. ND: not determined
